# Dual-use research and research using enhanced pathogens in high-income countries: whose business?

**DOI:** 10.1128/msphere.00168-24

**Published:** 2024-06-13

**Authors:** Nir Eyal, Melkizedeck Leshabari, Malabika Sarker

**Affiliations:** 1 Center for Population-Level Bioethics (IFH), HBSP (SPH), and Philosophy (SAS), Rutgers University, New Brunswick, New Jersey, USA; 2 School of Public Health and Social Sciences, Muhimbili University of Health and Allied Sciences, Dar es Salaam, Tanzania; 3 BRAC James P Grant School of Public Health, BRAC University, Dhaka, Bangladesh; 4 Heidelberg Institute of Global Health, Heidelberg University, Heidelberg, Germany; University of Michigan, Ann Arbor, Michigan, USA

**Keywords:** enhanced pathogens, dual-use research, biosecurity, biosafety, research governance, pandemic prevention, ethics

## Abstract

U.S. regulation of dual-use research of concern and of research with enhanced pathogens of pandemic potential may alter soon. Much has been written on the best form for that regulation to take. Less was written on a procedural question: whose voices should shape that regulation? This commentary addresses the latter, the procedural question, regarding the appropriate parties to the deliberations and decisions on this matter. It proposes to U.S. virologists that it would be in the interests of their discipline and only appropriate if that regulation were shaped by many voices from outside the discipline and from outside the United States.

## OPINION/HYPOTHESIS

U.S. oversight of biological research may soon undergo dramatic changes. Those pertain to dual-use research of concern (DURC)—research whose findings, useful as they may be, could facilitate the development of bioweapons; and research which creates or investigates enhanced pathogens of pandemic potential (ePPP).

The U.S. Office of Science and Technology Policy (OSTP), which may introduce these changes, seeks to balance enabling scientific studies that may generate new scientific and medical knowledge with another clear desideratum: preventing harmful lab leaks or findings that help malevolent actors. In the worst cases, those would result in new pandemic outbreaks. OSTP’s expected regulatory changes will follow a fall 2023 public commentary process on OSTP’s draft (1, 2), which heeded a related March 2023 report by the U.S. National Science Advisory Board for Biosecurity (3).

The pandemics that such regulation seeks to prevent would jeopardize all of us, not only the nations that fund or host studies that led to them (4–6). Indeed, they may especially jeopardize low- and middle-income countries (LMICs), where the bulk of the population can reside in close living quarters (7) and have other risk factors for severe outcomes. These countries could not possibly provide universal access to pandemic vaccines, therapeutics, and other expensive pharmaceutical and non-pharmaceutical interventions (e.g., to 222 nm far-UVC light [8], which in the foreseeable future will remain out of reach for most everyone at LMICs [9]), in part, because of richer nations’ vaccine nationalism, intellectual property laws, and wider economic policies. That is ironic because among these richer nations are the countries that, by conducting the bulk of global ePPP and DURC research, increase global pandemic risk. One might have argued that the medical advances make it worthwhile for LMICs to accept a somewhat augmented pandemic risk. But any medical advances that ePPP research may help develop, exciting as they may be (10), would typically remain unavailable for the vast majority of LMIC citizens for many years.

Even inside richer nations like the United States, vast health disparities put different income and racial groups at very different pandemic risks. And there too, any medical benefits from ePPP and DURC research may go to insured populations long before they reach everyone. All that makes it everyone’s business, or especially the business of LMICs and America’s and other rich nations’ racial and economic minorities, how U.S. research that may indirectly result in another pandemic is governed. The right conclusion is of course not that vaccines and other interventions should not be developed. Nor does our conclusion pertain to the first-order question of what regulations OSTP and decision-makers outside the United States ought to put forth in this area (4), given the international inequities (9). Our claim is, rather, that deliberation and decision-making in this area should involve global populations and their representatives. This is the most common ethical recommendation for any health research that affects broad and global populations (11–14). Our claim is also anchored in the general democratic principle that all those affected should have a say (15, 16).

In addition, balancing the benefits (10) and risks (17) from such research under appropriate weighting is complex. It involves not only considerations in virology and biosafety but also expertise in biosecurity strategy, global pandemic response, decision science, statistics, and population-level bioethics, to name just a few disciplines. It is therefore appropriate and welcome that OSTP’s September 2023 request for public input stated, “OSTP invites comment from any interested stakeholders” (1). The relevant experts hail from many academic disciplines; and when it comes to pandemics, people from every walk of life and from every country are relevant stakeholders.

Surprisingly, OSTP’s request for information then added:

In particular, OSTP is interested in input from research institutions, including both domestic and international entities, currently subject to the PC3O Policy or the DURC policies or that may be subject to the revised scope of a potential policy update, researchers within those institutions, scientific and professional organizations, and organizations representing diverse interests across the U.S. research ecosystem (1).

The stakeholders whose input is most emphatically sought here seemed to be the ones who have the greatest vested financial interest in the continuation of that research—institutions and individuals who are already steeped in that business. Some of the U.S. virologists commonly apprehensive about regulating the ePPP research (2, 10, 18) have direct financial stakes in the matter since it governs funding for their own studies. Curiously, in the area of ePPP research, it is routine to listen most acutely to the very scientists and research institutions who are conducting the same research that one is trying to oversee more effectively.

It is worth noting the peculiarity of what has informally become an expectation from discussants in this area—virological prowess. What about hearing first from experts in disciplines that have less direct financial stakes but pertinent and neglected input on this complex balance of governance, actuarial, and normative considerations? What about the voices of those nations, or U.S. population sectors, who would be at the highest risk from any pandemic that better oversight for U.S. (-funded) labs would have prevented, and who may benefit only modestly from the research that these labs pursue?

These added disciplinary and experiential inputs would protect the quality of decision-making in this important area, as well as public trust in virological science and its oversight. Currently, that trust is so low that political demagogues can score points with electorates by suppressing all so-called gain-of-function research (19), making no distinction between its concerning and perfectly justified (20) instantiations.

In that regard, the American Society for Microbiology’s traditional apprehensiveness about regulating this area comes at most members’ loss. Only a select few virology labs conduct ePPP and DURC research—most in no way benefit when its oversight remains limited. Most virologists and bacteriologists are net losers. Some have been convinced of an inevitable slippery slope to invasive regulation of many other areas of biological work, but there is a big difference between research with catastrophic potential and other, more mundane research. Having to defend research of castastrophic potential from careful oversight saddles the discipline with claiming expertise that virologists typically lack in many areas of this complex and multidisciplinary policy question, and with unwelcome public suspicion and political drama.

It is time to open this conversation more actively and purposefully to discussants from a wide range of personal, demographic, national, and disciplinary backgrounds and their representatives. None of us is safe until we can all have a strong voice on matters that, should they go wrong, as they might do (21), could upend global public health. That broader conversation is, among others, an interest of most U.S. microbiologists.

## ADDENDUM

During the proof stage for this paper, the OSTP efforts described matured into the U.S. Government Policy for Oversight of Dual Use Research of Concern and Pathogens with Enhanced Pandemic Potential. This important new policy renders the paper's findings all the more pertinent.
